# Development of a simple and highly sensitive virion concentration method to detect SARS-CoV-2 in saliva

**DOI:** 10.1016/j.heliyon.2024.e33168

**Published:** 2024-06-15

**Authors:** Yasuko Yamazaki, Uxía Alonso Alonso, Remil L. Galay, Wataru Yamazaki

**Affiliations:** a Center for Southeast Asian Studies, Kyoto University, 46 Shimoadachi-cho, Yoshida, Sakyo-ku, Kyoto, 606-8501, Japan; b Animal Medicine and Health at Institut de Recerca i Tecnologia Agroalimentàries (IRTA) – Centre de Recerca en Sanitat Animal (CReSA), Spain; c Department of Veterinary Paraclinical Sciences, College of Veterinary Medicine, University of the Philippines Los Baños, Los Baños, Laguna, 4031, Philippines; d Kyoto University School of Public Health, Konoe-cho, Yoshida, Sakyo-ku, Kyoto, 606-8303, Japan

**Keywords:** Concentration, COVID-19, SARS-CoV-2, Semi alkaline proteinase, Virion, Virus

## Abstract

**Background:**

Controlling novel coronavirus pandemic infection (COVID-19) is a global challenge, and highly sensitive testing is essential for effective control. The saliva is a promising sample for high-sensitivity testing because it is easier to collect than nasopharyngeal swab samples and allows large-volume testing.

**Results:**

We developed a simple SARS-CoV-2 concentration method from saliva samples that can be completed in less than 60 min. We performed a spike test using 12 ml of saliva samples obtained from healthy volunteer people, and the developed method performance was evaluated by comparison using a combination of automatic nucleic acid extraction followed by RT-qPCR detection. In saliva spike tests using a 10-fold dilution series of SARS-CoV-2, the developed method was consistently 100-fold more sensitive than the conventional method.

**Conclusions:**

The developed method can improve the analytical sensitivity of the SARS-CoV-2 test using saliva and speed up and save labor in screening tests by pooling many samples. Furthermore, the developed method has the potential to contribute to the highly sensitive detection of various human and animal viral pathogens from the saliva and various clinical samples.

## Highlights

• A method has been developed to detect SARS-CoV-2 from human saliva with 100 times higher analytical sensitivity.
• The developed method combines simple pretreatment within 60 min with conventional nucleic acid extraction and RT-qPCR.
• This method can be applied for more sensitive virus testing from individual saliva.
• This method can potentially be applied to screening more than 100 saliva samples.
• The method can be adapted to improve the analytical sensitivity of detecting pathogens from human and animal saliva.

## Abbreviations

ATCC: American Type Culture Collection
LOD: Limit of detection
PBS: Phosphate-buffered saline
PEG: Polyethylene glycol
RT-qPCR: Reverse transcription quantitative real-time polymerase chain reaction
SAP: Semi-alkaline proteinase
SARS-CoV-2: Severe acute respiratory syndrome coronavirus 2

## 1 Introduction

The COVID-19 epidemic continues as of 2023 and remains a public health threat (WHO). Virus detection using purified RNA obtained by extraction kits and real-time reverse transcription quantitative PCR (RT-qPCR) has been universally used due to its high analytical sensitivity and low incidence of false negatives due to low background [[1], [2], [3], [4]]. For environments with limited measurement resources, conventional RT-PCR-based testing methods have also been developed [3]. Virus detection using saliva has been used to diagnose respiratory infections such as COVID-19 and influenza because of its easy sampling and low burden on patients [[4], [5], [6]]. However, because of its somewhat lower analytical sensitivity compared to nasopharyngeal swab samples, false-negative results occur in samples collected early in infection or late in recovery when viral load is low, meaning that patients who slip through the test may not receive appropriate quarantine measures and become a potential source of infection [[4], [5], [6], [7]].

In addition, in the early stages of an epidemic, when there are few positive patients, it is crucial to test a large number of samples from a large number of people for negative confirmation to prevent the spread of infection [[8], [9], [10]]. In this case, to save cost and labor, pooling of samples, such as saliva [[8], [9], [10]], and nasal, nasopharyngeal and oropharyngeal swabs [9,[11], [12], [13]] from several people and testing them together is sometimes used for screening.

However, since the positive samples could be diluted by mixing with negative samples, leading to low virus concentration in the pooled sample, the test can become a false negative if it is below the limit of detection (LOD); hence, accurate detection may not be possible. As a solution, we have developed a method for detecting concentrated viruses in samples through immunomagnetic beads ([14];[15]. Still, it is not versatile because it requires specific antibodies for each virus.

The Polyethylene glycol (PEG) precipitation method has been used worldwide to enrich and detect norovirus and other viruses from oysters [[16], [17], [18]]. While this method can concentrate any virus, the presence of sample-derived inhibitors reduces concentration performance [[16], [17], [18], [19]]. In our previous studies, we have shown that a combination of a very short, low centrifugation process (900 *g*, 1 min) and a normal centrifugation process (10,000–20,000 *g*, 5 min) as a pretreatment step for simple concentration detection of target bacteria in chicken cecal contents (Sabike et al., 2016). Also, we have demonstrated that genetic testing for SARS-CoV-2 is possible without using an extraction kit by digesting human saliva containing potential genetic testing inhibitors with semi-alkaline protease (SAP) [7]. Here, we report the successful development of a new method for the concentration and detection of SARS-CoV-2 from a large volume of saliva by improving and integrating our previously published methods.

## 2 Materials and methods

### 2.1 Saliva sampling

Saliva samples were collected from three healthy volunteers, a female in her 40s, a female in her 20s, and a male in his 50s, all living in Kyoto, Japan, i.e., the three authors of this paper (YY, UAA, and WY), by repeatedly transferring drool collected in the oral cavity into a 50-ml sterile tube. After each saliva was thoroughly mixed by vortexing, the multiple saliva was promptly mixed in a new 50-ml tube to produce approximately 49 ml of pooled saliva. The three saliva samples used were confirmed to be SARS-CoV-2 negative by two RNA extraction methods (conventional and developed) and RT-qPCR detection, as described below, before the experiment.

### 2.2 Preparation of a 10-fold dilution series of SARS-CoV-2 spiked saliva

A 10-fold dilution series of heat-inactivated SARS-CoV-2 (ATCC VR-1986HK; American Type Culture Collection, Manassas, VA, USA) in PBS was prepared. The pooled saliva was dispensed into four or five 50-ml tubes of 12.2 ml each. SARS-CoV-2-containing saliva from neat to 10(−4) fold dilutions was prepared by sequentially spiking the 10-fold dilution series of SARS-CoV-2 into the 50 ml tubes containing pooled saliva and then vortexed thoroughly (Table 1).

**Table 1:** LOD determination of developed and conventional methods using saliva spiked with a 10-fold dilution series of SARS-CoV-2.

Test 1	Virus dilution	Neat	1:10	1:100	1:1000	1:10000
	Number of virus copies spiked per ml of saliva	1,00,000	10,000	1000	100	10
	Estimated virus number of copies per PCR reaction tube	28,800	2880	288	29	3
	Developed method	NT	31.507	**36.078**	No. Ct	No. Ct
	Estimated virus number of copies per PCR reaction tube	160	16	2	0.2	0.02
	Convetional method (PBS)	**38.211**	(38.967^a^)	No. Ct	No. Ct	NT
	Convetional method (SAP)	**37.526**	(38.694^b^)	No. Ct	No. Ct	NT
Test 2	Virus dilution	Neat	1:10	1:100	1:1000	1:10000
	Number of virus copies spiked per ml of saliva	40,000	4000	400	40	4
	Estimated virus number of copies per PCR reaction tube	11,520	1152	115	12	1
	Developed method	NT	36.163	**38.320**	(39.374^a^)	No. Ct
	Estimated virus number of copies per PCR reaction tube	64	6	0.6	0.06	0.006
	Convetional method (PBS)	**36.104**	(39.666^b^)	No. Ct	No. Ct	NT
	Convetional method (SAP)	**36.310**	No. Ct	No. Ct	No. Ct	NT
Test 3	Virus dilution	Neat	1:10	1:100	1:1000	
	Number of virus copies spiked per ml of saliva	20,000	2000	200	20	
	Estimated virus number of copies per PCR reaction tube	5760	576	58	6	
	Developed method	29.214	32.280	**38.089**	No. Ct	
	Estimated virus number of copies per PCR reaction tube	32	3	0.3	0.03	
	Convetional method (PBS)	**37.718**	No. Ct	No. Ct	No. Ct	
	Convetional method (SAP)	(38.933^b^)	(39.651^a^)	No. Ct	No. Ct	

LOD, Limit of detection.

No. Ct, No threshold cycle values detected using real-time PCR.

NT, Not tested.

Positive results were determined only when the Ct value was within 40.000.

LOD is shown in bold.

The figures in parentheses indicate one or two positive results out of three.

a , One positive in triplicate analysis.

b , Two positives in triplicate analysis.

### 2.3 RNA extraction by the conventional method

According to the pathogen detection manual 2019-nCoV issued by the National Institute of Infectious Diseases, Japan (NIID-J), two sets of the 100 μl saliva-spiked SARS-CoV-2 were collected into 1.5-ml microcentrifuge tubes and diluted 1:3 with 300 μl of PBS and sputum homogenizer SAP (Semi-Alkaline Proteinase, Suputazyme; Kyokuto Pharmaceutical Industrial, Tokyo, Japan), respectively. After sufficient vortexing, both (PBS and SAP) of the 1:3 dilutions containing SARS-CoV-2 were centrifuged at 20,000 *g* for 30 min, the former immediately and the latter after 15-min incubation with ten manual inversions mixing every 3 min at room temperature. The resulting 200 μl of the supernatant was transferred in a new 1.5-ml microcentrifuge tube and was set in an automated nucleic acid extractor MagLead 6 GC (Precision System Science, Co., Ltd, Matsudo, Japan) with MagDEA Dx SV reagent cartridge (Precision System Science) and were extracted and purified as RNA in 50 μl of distilled water.

### 2.4 Viral concentration and RNA extraction by developed method

**Fig. 1:**
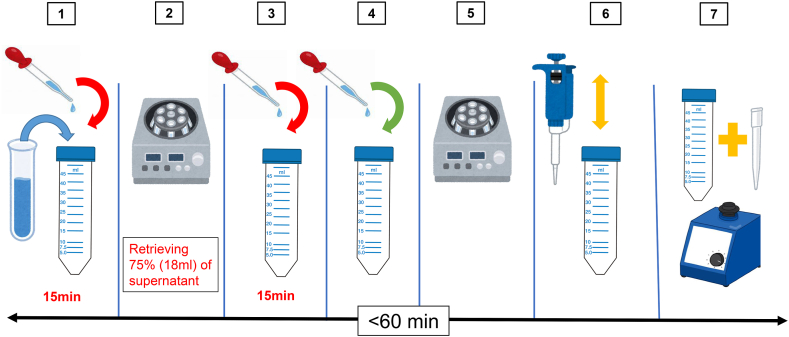
Developed protocol for virion concentration from saliva. SAP, Semi-alkaline proteinase. 1. Mix 12 ml of saliva with 12 ml of SAP (1:1) and keep for 15 min at room temperature. 2. After centrifugation at 4000 *g* for 5 min, carefully transfer 18 ml of the supernatant into a new 50-ml tube. 3. Add 15 ml of SAP to 18 ml of the supernatant, mix using a vortex and then keep at room temperature for 15 min. 4. After adding 13.2 ml of PEG-NaCl solution, mix by vortexing. 5. After centrifugation at 8000 *g* for 20 min, carefully discard the supernatant. 6. Add 100 μl of PBS and dissolve the invisible precipitates by pipetting and scraping with a 1-ml long tip (10 times each). 7. Place a 1-ml short tip into a 50-ml tube and vortex to completely dissolve the precipitate (supernatant residue after flushing + PBS ≒ 200 μl), and then transfer to an RNA extraction tube.

An overview is shown in Fig. 1. Specifically, 12 ml of SAP (Kyokuto) was added to the remaining 12 ml of saliva containing SARS-CoV-2. After vortexing, the mixture was kept at room temperature for 15 min. During the 15-min incubation, ten inversion mixings were performed manually every 3 min. Then, 4000 *g*, 5 min initial centrifugation was performed. Taking care not to inhale the pellet derived from the formed saliva components, 18 ml (75 % of the initial mixture volume) of the centrifugal supernatant was prudently transferred to a new 50-ml tube. 15 ml of SAP (Kyokuto) was added and incubated for 15 min at room temperature. Then, 13.2 ml of PEG solution (40 % PEG-NaCl, see details in our previous publication, [17]) was added and thoroughly mixed by vortexing, followed immediately by a second centrifugation at 8000 *g* for 20 min. The supernatant was carefully removed after the second centrifugation. To prevent contamination, 100 μl of PBS in a 1-ml long tip, which is longer than the 50 ml tube, was added. Pipetting was performed with the 1-ml long tip set pipet from the bottom of the tube to the area where the pellet had adhered during the first centrifugation, where precipitates of PEG-virus particle complex are assumed to be attached, although it cannot be seen with the naked eye. In addition, to completely detach any PEG-virus particle complex precipitates that may still be adhering to the tube wall, the 1-ml short tip was added to the 50-ml tube, the lid was closed, and the tube was thoroughly vortexed. Approximately 200 μl of the mixture of about 100 μl of PBS (containing PEG-virus particles) added to the around 100 μl of supernatant remaining on the inner wall of the 50-ml tube obtained by flushing was transferred into a 1.5-ml screw cap tube using a 1-ml long tip. The mixture was then extracted and purified as 50 μl of RNA using an automated nucleic acid extractor (Precision System Science), as described above. When the extracted RNA could not be tested immediately, it was stored at −80 °C until use.

### 2.5 Conducting RT-qPCR and determination of LOD

RT-qPCR was performed with 4 μl of the extracted RNA in 20 μl of the reaction mixture using a QuantStudio 3 (Thermo Fisher Scientific, Inc., Waltham, MA, U.S.A.), according to the method by Lu and colleagues (2020). The amplification time was slightly extended to ensure detection, as described below. Details of the reagents used are as follows: 20-μl RT-qPCR reactions comprised 10 μl of SuperScript III Platinum One-step RT-qPCR 2x reaction (Thermo Fisher Scientific), 0.4 μl of SuperScript III/Platinum *Taq* Mix (Thermo Fisher Scientific), 2 μl of primer (Hokkaido System Science Co. Ltd., Sapporo, Japan) probe (Integrated DNA Technologies, Inc, Singapore) mix for SARS-CoV-2 N2 detection reported by Emergency Use Authorization issued by the US Food and Drug Administration issued by the FDA [2], 2 μl of magnesium sulfate (50 mM, Thermo Fisher Scientific), 0.2 μl of Rox Dye (Thermo Fisher Scientific) diluted 1:5 with distilled water, 1.4 μl of nuclease-free water, and 4 μl of the RNA template. The cycling conditions were as follows: one cycle at 50 °C for 900 s and 95 °C for 120 s, followed by 50 cycles each at 95 °C for 15 s and 55 °C for 60 s. The automatically calculated Ct value was adopted, and the Ct cut-off value was set at 40.000. Positive results were determined if all three Ct values were within 40.000 in triplicate. Samples that showed only one or two positive Ct values in the triplicate analysis were interpreted as negative. The mean and standard deviation of the Ct were calculated for all samples interpreted as positive.

## 3 Results

Table 1, Table 2 and S-Table 1 show that the developed concentration method enabled 100-fold more sensitive detection than the conventional method by adding only a simple pretreatment within 60 min before the conventional extraction method. The developed method required only a centrifuge machine for 50 ml tubes up to 8000 *g* and a vortex for mixing the liquid in the 50 ml tubes and did not need expensive equipment such as an ultracentrifuge.

**Table 2:** Comparison of LOD between developed and conventional methods by RT-qPCR.

	LOD per ml of saliva	LOD per RT-qPCR reaction tube
Developed	200	58
Conventional (PBS)	20,000	32
Conventional (SAP)	40,000	64

As shown in Table 1, Table 2, the conventional method required 20,000 to 40,000 copies of virus per ml of saliva to detect SARS-CoV-2, whereas the developed method required only 200 copies of virus per ml of saliva. In other words, the developed method was at least 100 times more sensitive than the conventional method. Furthermore, a comparison of the number of viral copies per RT-qPCR reaction tube showed that 32 to 64 and 58 copies were required for detection by the conventional and developed methods, respectively. Namely, LOD per reaction tube was comparable between the developed method and the conventional method.

## 4 Discussion

In Japan, the airport quarantine for COVID-19 recommends collecting and submitting approximately 5 ml of saliva. Still, following the protocol of the NIID-J [20], 200 μl of the supernatant is generally used after diluting 200 μl within the range of 1:1 to 1:3 ratios and centrifuging at 20,000 *g* for 30 min. In other words, only 50–100 μl of saliva is used for the testing, and the remaining saliva of around 5 ml is used for nothing but retests. If all the unused samples were simply submitted to the concentration method developed in this study, the analytical sensitivity would be dramatically increased up to 100 times. Hence, a more accurate quarantine control measure would be possible.

Previously reports for detecting SARS-CoV-2 have succeeded in saving labor by pooling a large number of samples, but the analytical sensitivity is reduced because the positive samples are diluted ([8]; Praharaj et al., 2020[10]). Increasing the sample volume to 2 ml has succeeded in improving analytical sensitivity, but it lacks versatility because it relies on expensive specialized equipment [9]. In this study, we conducted a preliminary study to solve these problems by using a large amount of saliva (12 ml) to achieve both higher analytical sensitivity and labor savings. The disadvantage of pool testing is decreased LOD [6,8,10,12], but the developed method concentrates the trace amount of SARS-CoV-2, allowing for more sensitive detection than the conventional method or the previously reported method using pooled samples. Hence, the developed method is ideal for labor-saving large-scale screening in the early stages of an outbreak when the positivity rate is low.

**Fig. 2:**
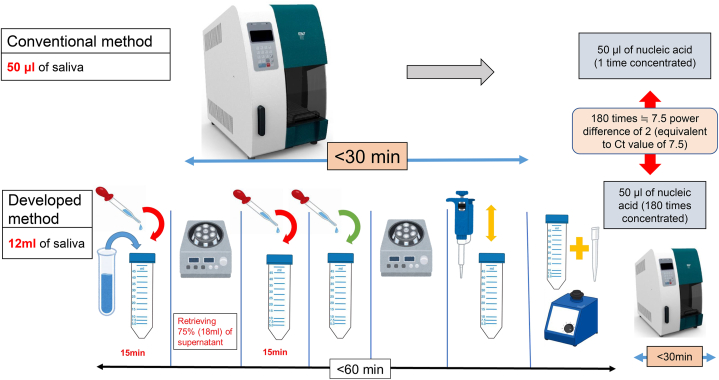
Virus detection from saliva with developed and conventional methods. SAP, Semi-alkaline proteinase. Conventional method: Nucleic acid extraction of 200 μl of centrifuged supernatant comprising of saliva 50 μl + PBS or SAP 150 μl (4x dilution). Developed method: Nucleic acid extraction after a simple concentration of 12 ml of saliva. The illustrations are cited from the following sources, all used in compliance with the terms and conditions. Pipettes and Micropipettes: Irasutoya (irasutoya.com). Vortex mixer: Kagaku Irasuto (science-illust.com). 50 ml tubes: Kenkyu Net (wdb.com/kenq/illust). Automatic nucleic acid extractor: Precision System Science Co. Ltd. (pss.co.jp/).

When a new variant emerges in the future, the concentration method could be used as a large-scale screening test to reasonably enhance quarantine control measures and contribute to efficient epidemic control. The developed method is theoretically capable of 180-fold virus concentration (Fig. 1, Fig. 2). Since the actual measured value is about 100-fold (Table 1, Table 2), it should be noted that the recovery rate could be reduced by approximately 50 %. In the conventional method, the 12-ml saliva sample is equivalent to 240 pooled samples of 50 μl of saliva per individual. Still, considering the recovery rate, a pool of more than 100 samples can be expected to have an analytical sensitivity equivalent to or better than that of the conventional method.

In our previous study, an immunomagnetic bead method using specific antibodies was successfully used to detect influenza A viruses added to PBS, duck feces, and chicken meat at 10- to 1000-fold sensitive concentration ([14]; [15]. Although this method is extremely sensitive, it is not very versatile because it requires the preparation of specific antibodies for each virus species and has the disadvantage that the LOD is reduced when the samples contain many inhibitory substances, such as components of the duck feces and chicken meat. In the present study, this problem has been successfully overcome by improving the pretreatment method with the combination of SAP, a sputum dissolving agent, and the PEG precipitation method to achieve highly sensitive SARS-CoV-2 concentration and detection in saliva.

The PEG precipitation method customarily includes an overnight 4 °C incubation process for virion capture. However, the NIHS protocol (2010; 20) states that the virion-PEG complex can be recovered from the oyster midgut gland immediately by centrifugation at 8000 *g* for 20 min without an overnight incubation. In the present study, we referred to this finding and confirmed that SARS-CoV-2 in saliva could be concentrated immediately after being centrifuged at 8000 *g* for 20 min without needing overnight incubation before the centrifuge, as expected. This allowed us to establish a rapid protocol successfully. This study's limitations include the inability to evaluate SARS-CoV-2-positive clinical samples and the fact that, although the concentration process is simple, the number of steps involved requires care by the examiner to avoid contamination and laboratory infection.

When the PEG-NaCl solution is mixed with a liquid sample containing trace amounts of virions, PEG, a polymer, adsorbs water molecules, causing the virions to aggregate. By centrifuging, the agglomerated virions-PEG complex can be precipitated on the wall of the tube. On the other hand, if the liquid sample contains impurities, this reaction is inhibited, and the recovery rate of virus particles is reduced. As shown in Table 1, the developed method is more sensitive than the conventional method, but the estimated number of copies of virus per reaction tube may be higher for the developed method to obtain a positive result. For example, as shown in Table 1, Table 2, Test 2 showed Ct values at 36.104 and 36.310 for the conventional method (64 copies/RT-qPCR reaction tube) versus the Ct value at 38.320 for developed method (115 copies/RT-qPCR reaction tube). This suggests that, although carefully collected, there is some residual material in the supernatant after centrifugation that inhibits virion-PEG complex formation or that there reduced viral recovery, resulting in an increase of the Ct value in the developed method.

In the developed method, only 75 % of the saliva centrifugal supernatant is used and the remaining 25 % must be discarded without use, as shown in Fig. 1, Process 2. In our preliminary experiment, the maximum amount of centrifugal supernatant corresponding to more than 90 % was collected, and concentration detection was attempted. However, contrary to expectations, the recovery rate was more than 10 times lower than the theoretical value (data not shown). In this case, a large pellet was identified on the tube by the naked eye in process 6 of Fig. 1. In other words, although the centrifugal supernatant appeared clear by the naked eye observation in Processes 2–5, we speculated that saliva components were mixed in and inhibited the formation of virion-PEG complexes. Therefore, we did not use the lower portion of the centrifugal supernatant, which was presumed to contain more saliva components due to the gradient caused by centrifugation but used only the upper 75 % to obtain a stable recovery rate.

Nevertheless, the developed method has the potential to solve the technical limitation of conventional genetic testing methods, i.e., the problem that samples carrying trace amounts of the virus have been judged as false negative because they are below the LOD [6,8,10,12,14]. For example, rabies is transmitted from dogs to humans via dog saliva. Still, definitive diagnosis requires dog brain emulsion containing large amounts of rabies virus, not the dog saliva, due to lack of the virus amount, and there is an animal welfare issue of euthanasia of dogs for sampling. The oral pulse oximeter used for anesthesia monitoring in veterinary clinics caused the transmission of severe fever thrombocytopenia syndrome (SFTS) virus transmission to cats due to contamination. However, the low amount of the virus was not detectable [21]. Both cases demonstrate the problem that current genetic testing cannot accurately detect trace amounts of virus in saliva, leading to false-negative diagnoses. We showed that this method has the potential to screen more than 100 saliva samples with power comparable to conventional extraction methods. In the future, this method may be applied to highly sensitive diagnosis of various human and animal viral infections that can be tested from saliva, as well as to rapid screening by pooled testing of a large number of samples.

## 5 Conclusions

We have successfully developed a simple and highly sensitive method for concentrating SARS-CoV-2 in saliva. The spike test demonstrated that the developed method has an extremely sensitive detection performance that is at least 100 times higher than the conventional method.

## Ethics approval

The study was conducted in accordance with the principles of the Declaration of Helsinki, and consent to participate was waived from three volunteer saliva sample donors who are also authors.

## Declaration of availability of data and materials

All data obtained in this study is included in the paper and Supplemental Table 1. In addition, the data sets in this study are available from the corresponding author upon reasonable request.

## Declaration of consent for publication

Consent for publication has waived the need to obtain informed consent from three volunteer saliva sample donors who are also authors.

## Funding

This research was supported by 10.13039/100009619AMED Number JP20he0622031, 10.13039/501100001691JSPS KAKENHI Numbers JP21H03180, JP22K05950, JP22KK0097, 10.13039/501100001691JSPS Bilateral Program Number JPJSBP120199944, and the Joint Usage/Research Center for Global Collaborative Research, Center for Southeast Asian Studies, 10.13039/501100005683Kyoto University.

## Data availability statement

Data included in article and supplemental (S-Table 1).

## CRediT authorship contribution statement

**Yasuko Yamazaki:** Writing – review & editing, Writing – original draft, Visualization, Validation, Resources, Methodology, Funding acquisition, Formal analysis, Data curation. **Uxía Alonso Alonso:** Resources, Methodology. **Remil L. Galay:** Writing – review & editing, Formal analysis. **Wataru Yamazaki:** Writing – review & editing, Writing – original draft, Visualization, Validation, Supervision, Resources, Project administration, Methodology, Funding acquisition, Data curation, Conceptualization.

## Declaration of generative AI and AI-assisted technologies in the writing process

During the preparation of this work, the authors used DeepL (DeepL SE, Cologne, Germany) to improve readability and language. After using this tool, the authors reviewed and edited the content as needed and took full responsibility for the content of the publication.

### Declaration of submission

The authors confirm that this manuscript or data has not been previously published and is not being considered for publication elsewhere. The authors further confirm that all authors have contributed to the study and have approved the final version.

## Declaration of competing interest

The authors declare that they have no known competing financial interests or personal relationships that could have appeared to influence the work reported in this paper.
